# Bidirectional rotating biatrial tachycardia

**DOI:** 10.1002/joa3.12919

**Published:** 2023-08-25

**Authors:** Koichiro Yamaoka, Tomoyuki Arai, Masao Takahashi, Rintaro Hojo, Seiji Fukamizu

**Affiliations:** ^1^ Department of Cardiology Tokyo Metropolitan Hiroo Hospital Tokyo Japan

**Keywords:** ablation, atrial tachycardia, biatrial tachycardia, bidirectional rotation, epicardium

## Abstract

A 72‐year‐old man was treated for recurrent atrial tachycardia (AT) and underwent ablation. The AT was diagnosed as bi‐AT based on the activation map and the postpacing interval. Another AT appeared and was diagnosed as bi‐AT by the same method. Surprisingly, the circuits of both ATs were perfectly matched and rotated in opposite directions. The left atrial anteroseptal wall was ablated during the AT. The AT was immediately stopped and was no longer induced.

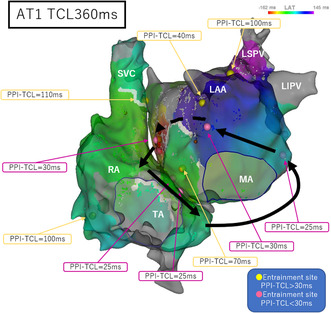

Several studies have reported on circuits of biatrial tachycardia (bi‐AT), including one report summarizing several circuits of bi‐AT.^1^ However, there are no reports of sufficient evaluation of bidirectionally rotating bi‐ATs, in which two atrial tachycardias (AT) have the same circuit and rotate in opposite directions. Herein, we report a case of bidirectional bi‐AT rotating on the same circuit that was diagnosed by mapping and an electrophysiological study.

A 72‐year‐old man who had undergone aortic valve replacement developed heart failure 5 months before presentation, during which atrial fibrillation (AF) was first documented. Three months prior to his visit, he underwent ablation (pulmonary vein and left atrial posterior wall isolation) for persistent AF. At the time of examination, he had recurrent AT and underwent ablation.

At the start of ablation, the patient's arrhythmia was AF; therefore, defibrillation was performed and the AF was stopped. We obtained a voltage map of the left atrium during right atrial pacing using a 3D mapping system (CARTO; Biosense Webster), which confirmed the isolation of the pulmonary vein and left atrial posterior wall. A broad low voltage zone (LVZ) was observed in the left atrial anterior wall (Figure 1). Because this LVZ may have been involved in AT recurrence, we ablated the area on the surface.

**FIGURE 1:**
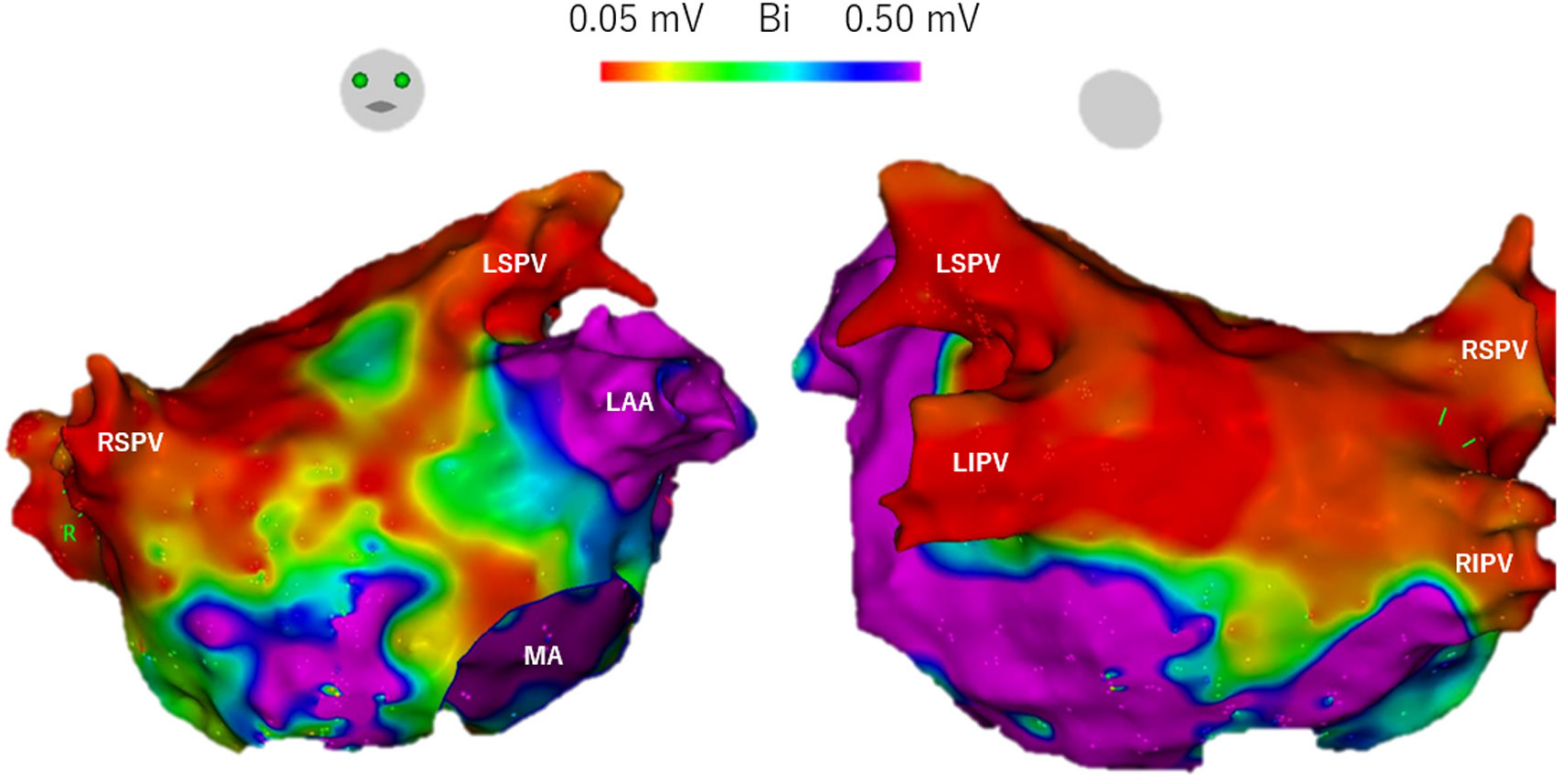
Left atrial voltage map, anterior–posterior (left) and posterior–anterior (right) views. The pulmonary vein and left atrial posterior wall were isolated. A broad low voltage zone was observed in the left atrial anterior wall. LAA, left atrial appendage; LIPV, left inferior pulmonary vein; LSPV, left superior pulmonary vein; MA, mitral annulus; RIPV, right inferior pulmonary vein; RSPV, right superior pulmonary vein.

After the ablation, an AT with a tachycardia cycle length (TCL) of 360 ms was induced by pacing (AT1). We mapped the left atrium during AT1, but the local activation time (LAT) did not meet the TCL of AT1. We also mapped the right atrium during AT1, and the LAT of the bilateral atrial maps met the TCL of AT1. The postpacing intervals (PPIs) of the mitral annulus at the 3 o'clock position, base of the left atrial appendage, left atrial anteroseptal wall, right atrial septum, and entrance of the coronary sinus (CS) were similar to the TCL of AT1 (Figure 2). The PPI of the lower left atrial anterior wall near septum was 70 ms longer than TCL of AT1 (Figure 2), which ruled out the possibility that AT1 was left atrial AT using the left atrial septum and lower anterior wall. Based on the PPI and biatrial activation map, we diagnosed AT1 as a bi‐AT with the left atrial anterior wall, right atrial septum, and CS as a counterclockwise rotating circuit (Video 1).

**FIGURE 2:**
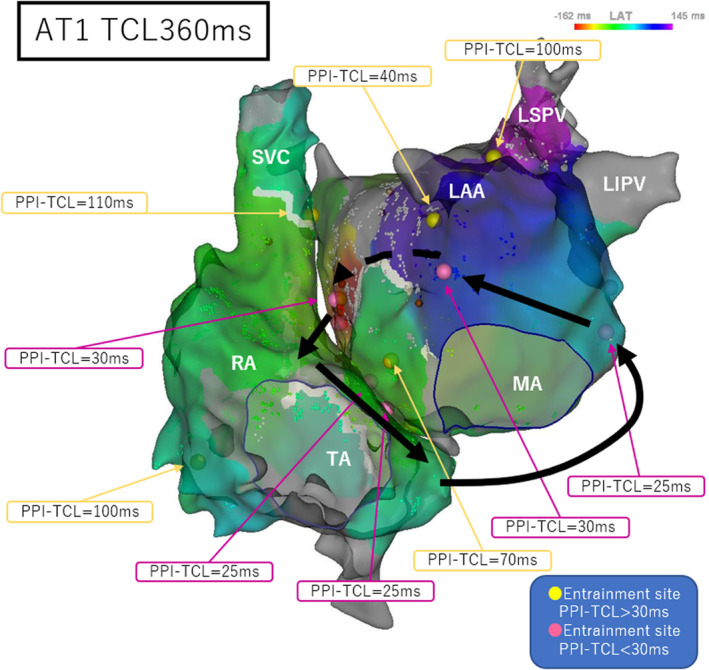
Bilateral atrial activation maps and PPI for AT1 (AT with a TCL of 360 ms induced by pacing after ablation). The black arrows indicate the assumed circuits of AT1, and the dashed line indicates the assumed epicardial circuits. AT, atrial tachycardia; LAA, left atrial appendage; LAT, local activation time; LIPV, left inferior pulmonary vein; LSPV, left superior pulmonary vein; MA, mitral annulus; PPI, postpacing interval; RA, right atrium; SVC, superior vena cava; TA, tricuspid annulus; TCL, tachycardia cycle length.

**VIDEO 1:** Bilateral atrial activation maps for AT1 and AT2. These ATs bidirectionally rotated the circuit with the left atrial anterior wall, right atrial septum, and CS. AT, atrial tachycardia; CS, coronary sinus.

We stopped AT1 by pacing to evaluate the conductivity of the left atrial anterior wall, where the LVZ extended. We performed differential pacing, but could not evaluate the conductivity because of the extremely wide LVZ. Another AT with a TCL of 360 ms was induced by pacing (AT2). Because the LAT of the left atrial mapping did not meet the TCL of AT2, we mapped the bilateral atrium and the LAT of the bilateral atrial maps met the TCL of AT2. The PPIs of the mitral annulus at the 1 o'clock position, base of the left atrial appendage, left atrial anteroseptal wall, right atrial septum, and CS entrance were similar to the TCL of AT2 (Figure 3). Together with the activation map, we diagnosed AT2 as a bi‐AT.

**FIGURE 3:**
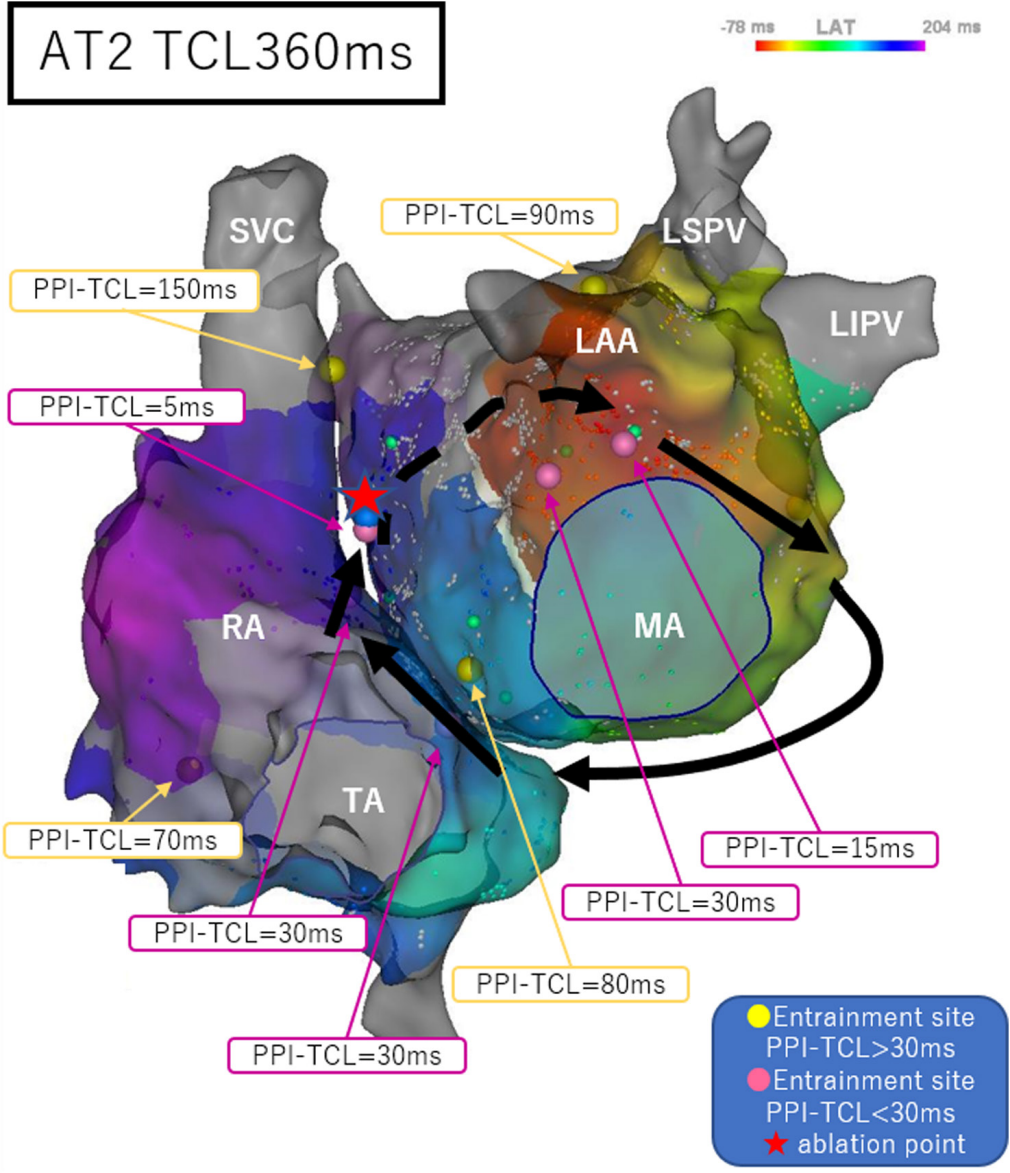
Bilateral atrial activation maps and PPI for AT2 (another AT with a TCL of 360 ms induced after reinduction by pacing). The black arrows indicate the assumed circuits of AT2, and the dashed line indicates the assumed epicardial circuits. The red star represents the ablation point. AT, atrial tachycardia; LAA, left atrial appendage; LAT, local activation time; LIPV, left inferior pulmonary vein; LSPV, left superior pulmonary vein; MA, mitral annulus; PPI, postpacing interval; RA, right atrium; SVC, superior vena cava; TA, tricuspid annulus; TCL, tachycardia cycle length.

AT2 rotated the same circuit as AT1, only in the opposite direction (Video 1). In addition, there was an area near the lower middle of the left atrial anterior wall where the PPI was not similar (Figure 2). These findings indicated that the left atrial anterior wall may partially pass through the epicardium. We ablated the left atrial anteroseptal wall during AT2, and AT2 stopped at about 30 s after the first ablation. Thereafter, AT was no longer inducible.

Several studies have reported on circuits of bi‐AT, including one reporting that the atrial septum, CS, and Bachmann bundle were used as circuits.^1^ However, there are no reports of sufficient evaluation of bidirectionally rotating bi‐ATs. The causes of bi‐AT include ablation of the left atrial anterior line^2^ and mitral valve surgery.^3^ In our case, neither was present; however, the LVZ had spread to the left atrial anterior wall, which was likely the critical isthmus. We also assumed that part of the left atrial anterior wall passes through the epicardium and that the only site where the left atrial anterior wall can be ablated from the endocardium is at the left atrial anteroseptal wall or the base of the left atrial appendage, which is the endocardial‐epicardial junction. Therefore, the anteroseptal wall of the left atrium was ablated.

Possible reasons for the lack of similar reports are as follows. First, ablation is almost always started once the circuit is identified. In this case, we were able to detect bidirectional bi‐AT because we stopped the AT once to evaluate the conductivity in the left atrial anterior wall. Next, the conduction pattern of the critical isthmus in the bi‐AT circuit may be related. However, this remains unclear and requires further study.

We report the first case of a bidirectional rotating bi‐ATs in the same circuit, which may help to clarify the properties of AT circuits including critical isthmus.

## CONFLICT OF INTEREST STATEMENT

The authors declare no conflicts of interest.

## DECLARATIONS

*Approval of the Research Protocol*: Not applicable.

*Informed Consent*: Acquired.

*Registry and Study Registration No*: Not applicable.

*Animal Studies*: Not applicable.
